# Biocompatible Customized 3D Bone Scaffolds Treated with CRFP, an Osteogenic Peptide

**DOI:** 10.3390/bioengineering8120199

**Published:** 2021-11-30

**Authors:** Vamiq M. Mustahsan, Amith Anugu, David E. Komatsu, Imin Kao, Srinivas Pentyala

**Affiliations:** 1 Department of Anesthesiology, Stony Brook University, Stony Brook, NY 11794, USA; vamiqmohammed.snu@stonybrook.edu (V.M.M.); Amith.Anugu@stonybrook.edu (A.A.); 2 Department of Mechanical Engineering, Stony Brook University, Stony Brook, NY 11794, USA; Imin.Kao@stonybrook.edu; 3 Department of Orthopedics, Stony Brook University, Stony Brook, NY 11794, USA; David.Komatsu@stonybrook.edu

**Keywords:** osteogenic peptide, CRFP, artificial bone, ABS, MED610, scaffolds, 3D printing

## Abstract

Background: Currently used synthetic bone graft substitutes (BGS) are either too weak to bear the principal load or if metallic, they can support loading, but can lead to stress shielding and are unable to integrate fully. In this study, we developed biocompatible, 3D printed scaffolds derived from µCT images of the bone that can overcome these issues and support the growth of osteoblasts. Methods: Cylindrical scaffolds were fabricated with acrylonitrile butadiene styrene (ABS) and Stratasys^®^ MED 610 (MED610) materials. The 3D-printed scaffolds were seeded with *Mus musculus* calvaria cells (MC3T3). After the cells attained confluence, osteogenesis was induced with and without the addition of calcitonin receptor fragment peptide (CRFP) and the bone matrix production was analyzed. Mechanical compression testing was carried out to measure compressive strength, stiffness, and elastic modulus. Results: For the ABS scaffolds, there was a 9.8% increase in compressive strength (*p* < 0.05) in the scaffolds with no pre-coating and the treatment with CRFP, compared to non-treated scaffolds. Similarly, MED610 scaffolds treated with CRFP showed an 11.9% (polylysine pre-coating) and a 20% (no pre-coating) increase (*p* < 0.01) in compressive strength compared to non-treated scaffolds. Conclusions: MED610 scaffolds are excellent BGS as they support osteoblast growth and show enhanced bone growth with enhanced compressive strength when augmented with CRFP.

## 1. Introduction

The development of bone graft substitutes (BGS) is an essential field of orthopedic research [1]. BGS are usually used to fill the bone defects formed as a result of surgical procedures, fractures, or oncology [2,3,4]. In the majority of cases, the BGS used are either autografts or allografts. Historically, autografts have been considered the gold standard in bone grafting, as they have a lower probability of disease transfer, are osteoconductive, and allow for osteogenesis [5,6]. However, an autograft has many disadvantages, as the graft is extracted from another site in the body, resulting in excess pain in the donor site, surgical and wound complications, low graft availability, and increased hospital stay [7,8,9,10]. Allogenic bone grafts or allografts are the most commonly used BGS, as they are also osteoconductive and avoid donor site complications. However, they have their range of disadvantages, such as lack of uniformity, a higher chance of antigen response and disease transfer, and limited supply [11,12]. Additionally, sterilized allografts are usually weaker than healthy bone, increasing the graft site’s risk of failure and reinjury [7,13].

Synthetic BGS are being developed to deal with the complications of autografts and allografts [14]. One example is demineralized bone matrix (DBM) which is derived by removing the mineral components of the allogenous bone with acid, leaving behind collagen and endogenous bone growth factors [15,16,17]. This process makes DBM a good osteoinductive material and also promotes osteoconductivity; however, it reduces the strength of the original bone [18,19]. DBM also has a higher rate of disease transmission from the host bone [6]. Ceramic grafts are another class of BGS that are derived based on calcium salts and their variants [4,14]. Ceramic BGS do not have a limitation in quantity, can be easily sterilizable, and do not risk morbidity or disease transmission. However, they have disadvantages as they have a high resorption rate, which may not lead to complete bone formation and may cause weakening of the bone [20,21]. It may also stimulate an inflammatory response due to elevated levels of particulate disintegration products [22]. The applications of ceramic BGS are limited to non-load-bearing bone defects as they cannot provide the structural support needed by the bone in the long term [23]. The other option is using metallic implants, which are strong and can provide structural support in the bone. However, they do not allow for osteoconductivity or osteointegration in the bone [24,25,26]. Metallic implants are extensively being used in long bone arthroplasty and limb salvage applications. However, the higher strength of these implants than the surrounding bone causes stress shielding, which weakens the surrounding bone [27,28]. These complications may lead to major revision surgeries in the future and affect the quality of life by reducing the ease of functionality of the patient.

Three-dimensional (3D) printing or additive manufacturing (AM) is an exciting new advance that has helped in improving ease and accuracy in the fabrication of complex and customized shapes. Owing to the ability to control the porosity and permeability in the design of the BGS, many studies have used 3D printing in developing implants [29,30,31]. Selective laser sintering (SLS) is a 3D printing technique used to 3D print patient-specific customized metallic implants that are widely being used in limb arthroplasty procedures. SLS is also being used in fabricating ceramic BGS by fusing layer-on-layer of ceramic material [32,33]. Konopnicki et al. seeded bone marrow progenitor cells (pBMP) on the surface of 3D printed ceramic polycaprolactone (PCL) BGS and showed improved biocompatibility and bone penetration into the implant [34,35]. Fused deposition modeling (FDM) is a 3D printing technique to fabricate layered structures using thermoplastic materials extruded through a heated nozzle [36]. Many studies have been carried out using FDM-fabricated BGS which include polyglycolic acid (PGA), polylactic acid (PLA), and their co-polymers polylactic co-glycolic acid (PLGA) and their combinations with ceramic materials [37,38,39]. These polymers are biodegradable and form lactic acid which dissolves in the body [40]. However, these scaffolds have not yet achieved the results required for application in the current clinical practice [41]. The main reason for this is high resorption rate which reduces the mechanical strength of the scaffold with time and makes them unsuitable for load-bearing sites [22]. In spite of these advances, it has still been a challenge to develop ideal BGS for load-bearing sites, which are osteoconductive, osteoinductive, allow for osteointegration, and at the same time provide the surrounding bone with strength comparable to that of the healthy bone.

Helguero et.al. developed 2D acrylonitrile butadiene styrene (ABS) scaffolds fabricated using FDM printing, pre-treated with calcitonin receptor fragment peptide (CRFP) and showed enhanced biomechanical characteristics and bone matrix proliferation [42]. The ABS material is a non-biodegradable polymer which has shown to be biocompatible and allows for osteoconduction on its surface [42,43]. This gives ABS the advantage of maintaining its structural properties and also allows for the bone to integrate into the scaffold. CRFP, an active peptide domain on the C-terminus of the calcitonin receptor [44,45], was found to be bioactive and osteoinductive in its nature to enhance bone matrix production and induce differentiation of stem cells into osteoblasts [35]. In vivo studies show that CRFP increases bone matrix production when used along with other osteogenic reagents [44,45].

Stratasys^®^’s MED610 material is also a non-biodegradable material which has shown biocompatibility compliance with ISO 10993 [46]. MED610 is fabricated using the polyjet printing process which generates layers of the 3D model by solidifying a photosensitive polymer resin [47,48]. The polyjet printing process results in high-resolution products (<20 μm) in comparison to conventional FDM printing (200 μm). This material has great potential to enhance the flexibility and accuracy in developing a BGS with trabecular structural patterns as well as improve precision in the scaffold production.

In this study, we developed and evaluated the biocompatibility, osteoconductivity, and biomechanics of ABS and MED610 bone scaffolds inspired by natural bone’s structural physiology, seeded with MC3T3 stem cells, and treated with CRFP.

## 2. Materials and Methods

### 2.1. Design and Fabrication of Cylindrical Scaffolds

**Figure 1:**
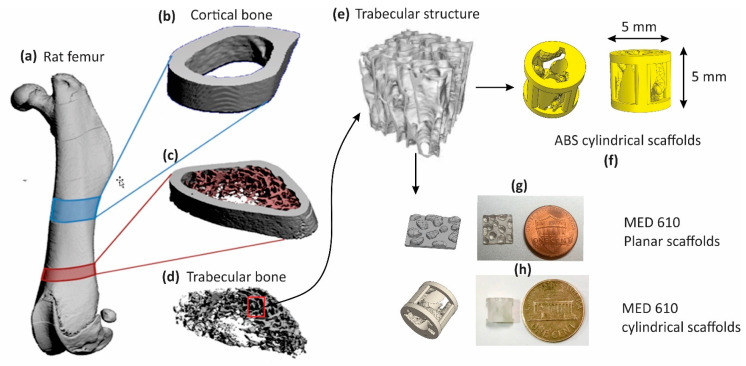
(**a**) Three-dimensional CAD model of a rat femur with specified regions for cortical bone (blue) and trabecular bone (red). (**b**) Cross-sectional region of cortical bone, R1 and (**c**) trabecular bone, R2. (**d**,**e**) The extraction of the trabecular pattern and (**f**) structures of ABS (cylindrical) and (**g**,**h**) MED610 implants (cylindrical and planar implants) alongside a penny for scale.

A femur of a skeletally mature rat was computerized tomography (CT)-scanned (μCT40, Scanco medical) at an isotropic voxel resolution of 10 μm. The resulting CT imaging files (DICOM files) were converted into a 3D stereolithography model (STL file) by thresholding and combining the DICOM files using InVesalium software (Renato Archer Information Technology Center, Brazil). This 3D representation was processed in Geomagic^®^ DesignX software. Regions of interest were identified to extract the cortical bone structure (R1) from the diaphysis and the trabecular structure (R2) at the metaphysis of the femur assuming a segmental defect model, as illustrated in Figure 1b,d These structures (R1 and R2) were converted into a computer-aided design model (CAD file) using a non-uniform rational B-spline (NURBS)-based algorithm and uploaded into Solidworks^®^ software. We designed a cylindrical scaffold (5 mm height × 5 mm diameter) with internal structures created from the trabecular patterns extracted from R2 and four pillar structures with equal spacing on the outer rim of the cylinder, having dimensions (1 mm thickness × 1.5 mm length × 5 mm height) derived from the cortical bone thickness in R1. We carried out many reiterations by extracting different trabecular features and porosity and finalized our design based on the structural consistency of the 3D-printed result. Our final design had a porosity of 50% to facilitate penetration of the cells into the scaffold. Three-dimensional printing gives us the flexibility to change the number of pillars or the density of the trabecular structures based on the location and the strength requirements in the implantation site. Using the finalized design, we 3D-printed ABS scaffolds using FDM fabrication (Cube Pro Duo printer, 3D Systems-200 μm resolution, Rock Hill, SC, USA), and MED610 scaffolds were fabricated using polyjet 3D printing (Objet 30 Prime-16 μm resolution, Stratasys^®^, Eden Prairie, MN, USA) [46]. We also developed planar (2D) scaffolds (10 mm × 10 mm) of MED610 material to test the osteoconductivity of the material surface.

### 2.2. Preparation of the Scaffolds

The ABS scaffolds were washed with deionized water for 24 h, followed by sterilization in an increasing concentration of ethanol solution (50%, 70%, 80%, 90%, and 100%) for 15 min and air-drying in a cell culture hood. The MED610 scaffolds were also washed in deionized water. However, sterilization was done by autoclaving at 132 °C for 4 min as per the manufacturer’s recommendation.

We pre-coated the scaffolds by treating them with 0.01% polylysine solution for 2 h, rinsing them with sterile Dulbecco’s phosphate buffer saline (DPBS), and air-drying for 1 h in a sterile cell culture hood. Polylysine, a known contributor to osteoconduction, has been shown to help the cells to attach to the scaffold surface [49,50].

### 2.3. Seeding MC3T3 Cells

After the sterilization and pre-coating (in some cases), the scaffolds were attached to the bottom surface of the cell culture plate using sterilized vacuum grease to prevent them from floating during the cell culture process. MC3T3 cells extracted from the C3 vertebra of a mouse with a density of 1 × 10^3^ cells per chamber were seeded onto the scaffolds and cultured in media (MEMα supplement with 5% fetal bovine serum and 1% penicillin/streptomycin) [51]. The culture media was changed every three days.

### 2.4. Bone Matrix Deposition on the 3D-Printed Scaffold

After the cells reached 80% confluency, we added 4 mM β-glycerol phosphate (G6P) and 0.05 μg/μL ascorbic acid (AA) to induce osteogenesis in the cells [52,53]. The cells on the scaffolds were further cultured for 21 days for the bone matrix to proliferate on the surface. The culture media, along with osteogenic agents, was changed every three days. After 21 days, the hybrid scaffolds were incubated in 4% formaldehyde solution (Electron Microscopy Sciences, Hatfield, PA, USA) for 30 min at room temperature to fix the bone matrix on the scaffold. After that, the scaffolds were washed and stored in DPBS solution.

### 2.5. Osteoconductivity of MED610 Scaffolds

The MED610 material is a USP Class VI standard biocompatible material and is certified not to cause an inflammatory response with implantation [54]. To investigate its feasibility as a suitable material for developing our BGS, we evaluated the osteoconductivity, i.e., cell growth and bone matrix deposition on the surface of MED610. We seeded the planar scaffolds with MC3T3 stem cells and induced osteogenesis, as explained in Section 2.3 and Section 2.4. We evaluated the cell growth by carrying out scanning electronic microscopy imaging (10.0 Kx magnification at electron high tension (EHT) voltage of 2.5 kV). The von Kossa staining method was applied to assess the bone matrix deposition on the scaffold surface. Ten regions of interest (ROI) (1 mm × 1 mm) were identified on the planar scaffolds. The images of these ROI for seeded and unseeded scaffolds were taken after the von Kossa staining. These images were processed in Matlab^®^ and the pixel values were normalized with ‘0’ being the brightest measure (100% white) and ‘1’ being the darkest measure (100% black). We calculated the average of these normalized pixel intensities for each ROI as a measure to quantify dark spots on the scaffold (representing calcium salts from the bone matrix deposition). A von Kossa image with bone matrix deposition on the scaffold surface will have darker pixels in the ROI, and average dark pixel intensities would be higher in comparison with the scaffold surface without bone matrix deposition. Comparative *t*-tests at a significance level of 0.05, were carried out to determine the difference in average dark pixel intensity of ROIs of unseeded and seeded scaffold surfaces.

### 2.6. Mechanical Properties of Scaffolds

**Figure 2:**
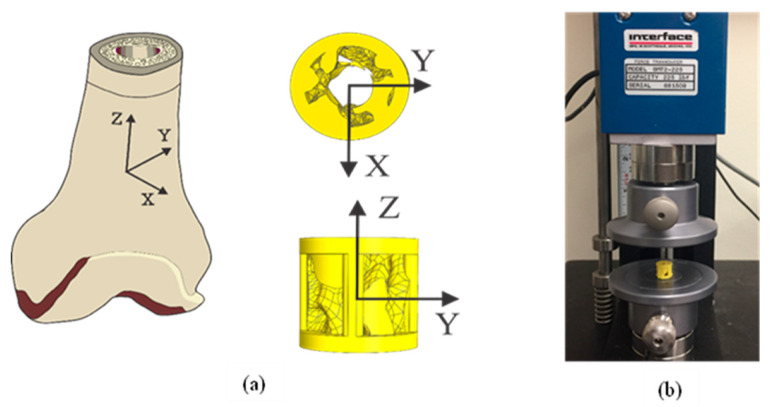
(**a**) The three principal axes of the femur bone with the Z-axis being the longitudinal axis; X-axis is the sagittal axis and Y-axis is the frontal axis. The cylindrical axis of the implant is the longitudinal axis. (**b**) MTEST Quattro compressive testing apparatus for the mechanical tests.

As the cylindrical scaffolds we designed were inspired by the cortical bone and trabecular bone (R1 and R2) in a segmental defect model (Figure 1), ideally, they should follow similar mechanical properties as demonstrated by a healthy bone. Mechanical compressive testing (MTEST Quattro, Admet, Norwood, MA, USA) was performed on ABS and MED610 scaffolds to study the anisotropy of the scaffolds. Ten (n = 10) compressive tests in each direction were carried out in the three orthogonal axes with the Z-axis as the longitudinal axis, as illustrated in Figure 2. The scaffolds were placed in between the two stainless steel plates, and unconstrained compression was carried out, as shown in Figure 2b. The speed of measurement was maintained at 5 mm/min based on ISO 604 convention for measurement of plastics [55]. Mechanical characteristics including stiffness (k), maximum compressive strength ($\sigma_{M}$), and compressive modulus ($E_{C}$) were calculated in each axis and compared to get a measure of anisotropy [56]. From previous studies, the relationship between the maximum compressive stress about the three axes in a femur bone is expressed as Equation (1) [57]. This relationship indicates that along the Z-axis (longitudinal axis), the femur bone shows better strength in comparison to the other orthogonal axes.

(1) $$\;\sigma_{Longitudinal\;axis}>\sigma_{Sagittal\;axis}\geq \sigma_{Frontal\;axis}$$

### 2.7. In Vitro Study and Strength Analysis of Cylindrical Scaffolds Treated with CRFP

As explained earlier, ideal BGS need to have similar strength characteristics to that of the natural bone, be osteoconductive (allow for bone growth), and be biocompatible (accepted by the host). To achieve this, we seeded MC3TC cells onto the cylindrical scaffolds and induced osteogenesis to generate bone matrix. This layer of bone matrix has been shown to improve the osteoconductivity of the BGS after implantation and helps in bone penetration into the implant [58]. It has also been shown that, in addition to helping the scaffold better integrate with the host bone, the bone matrix also enhances the strength of the scaffold [42]. We carried out studies with ABS and MED610 cylindrical scaffolds (n = 10), with varying protocol combinations of scaffold preparation and osteoinduction to generate more bone matrix deposition. Many studies suggested that pre-coating the scaffolds with polylysine helps in promoting the growth of bone matrix as it helps the cells to adhere to the surface [49].

CRFP, which is known to enhance the bone matrix production and contribute to osteoinduction, was actively used for inducing osteogenesis along with other osteogenic agents (ascorbic acid and glycerol phosphate). For the ABS and MED610 scaffolds, the different combinations for pre-coating and osteoinduction are listed in Table 1 and Table 2, respectively. After inducing osteogenesis for 21 days and fixing the scaffolds with 4% formaldehyde, the scaffolds were evaluated for bone matrix deposition by compressive testing to evaluate the maximum compression strength ($\sigma_{M}$), stiffness (K), and the compressive modules ($E_{c}$). Thereafter, we carried out the Shapiro–Wilk test for the compressive test results of each type of scaffold to assess the normality of the data before carrying out the statistical analysis.

For the ABS material, we carried out a one-way analysis of variance (ANOVA) at a significance level of 0.05 to compare the changes in mechanical properties of scaffolds pre-treated with polylysine to the original scaffolds (Types A1, A2, and A3). In addition, we carried out a Student’s *t*-test to compare the original scaffold to the scaffolds with no pre-coating (Types A1 and A4).

For MED610 material, we carried out two one-way ANOVA tests at a significance level of 0.05. The first one-way ANOVA test involved scaffolds pre-treated with polylysine being compared to the original strength of the scaffold (Types M1, M2, and M3). In the second test, we compared the scaffolds with no pre-coating (Types M1, M4, and M5). We followed up the one-way ANOVA tests with a pairwise comparison of the groups by carrying out Student’s *t*-tests at a significance level of 0.017 (Bonferroni correction).

Even though the 3D structure and porosity of the ABS and MED 610 scaffolds are similar, the intrinsic mechanical properties of these materials differ, which leads to varying results in compression testing of both materials. To compare the performance of ABS with MED 610 scaffolds, we evaluated the structural contribution of bone matrix in enhancing the strength of the scaffolds in both materials by subtracting the strength of seeded and differentiated scaffolds with the mean strength of original unseeded scaffolds. We compared similar types in ABS and MED 610 by carrying out a Student’s *t*-test.

Post-compression, ABS scaffolds were stained with 2% Alizarin red (Lifeline Cell Technologies) for validating calcium deposits (a mark of bone deposition) and imaged (Zeiss LSM-510). Imaging studies were carried out as explained in Section 2.5, and the residual red spots formation (post-staining) representing calcium deposits were evaluated by calculating the average red color intensity values. Student’s *t*-tests at significance level of 0.05 were carried out to compare the calcium deposition in different types of ABS scaffolds.

Similarly, in MED610 scaffolds, the von Kossa staining method was applied to validate the bone matrix deposition on the scaffold surface and an imaging study was carried out as explained in Section 2.5.

## 3. Results

### 3.1. Bone Matrix Deposition on MED610 Planar Scaffolds

**Figure 3:**
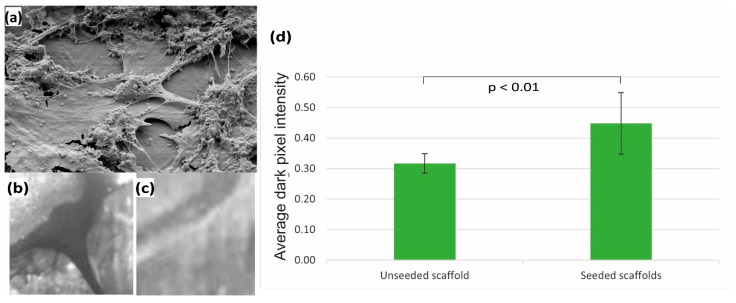
(**a**) Scanning electron microscopy imaging at 10 KX magnification on the planar scaffolds, taken 21 days after osteoinduction, showing proliferation of cells and cell matrix on the scaffold; (**b**) region of interest (ROI) image of von Kossa staining for planar scaffolds seeded with bone matrix (darker area representing calcium salts) and (**c**) corresponding ROI image for unseeded planar scaffolds. (**d**) Results showing the increase of average dark pixel intensities on the seeded scaffolds in comparison to unseeded scaffolds after von Kossa staining (n = 10). Error bars represent the standard deviation.

From the scanning electron microscopy studies on planar scaffolds, we see that there is indeed bone growth on the surface of MED610, as the cells attached to the surface are shown in Figure 3a. The von Kossa staining study to evaluate the deposition of bone matrix shows a significant increase in the average dark pixel intensity (*p* = 0.001), demonstrating significant mineralization on the surface of MED610 scaffolds seeded with bone cells in comparison to unseeded scaffolds, showing that it is an osteoconductive material and allows for bone matrix deposition (Figure 3).

### 3.2. Mechanical Properties of the Cylindrical Scaffolds

**Figure 4:**
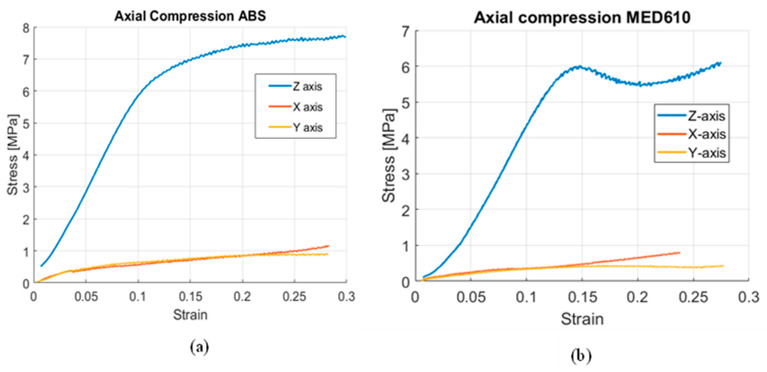
(**a**) The stress–strain curve in the three principal axes for ABS scaffolds, and (**b**) the stress–strain curve of the MED 610 scaffolds in the three principal axes.

The compression studies of ABS and MED610 scaffolds are expressed in the stress vs. strain plots in Figure 4a,b, respectively. These results follow a similar trend that we observe in the literature for healthy bone, where the scaffolds exhibit higher strength in the direction of the longitudinal axis, and the other two orthogonal axes performing at considerably lower strength.

**Figure 5:**
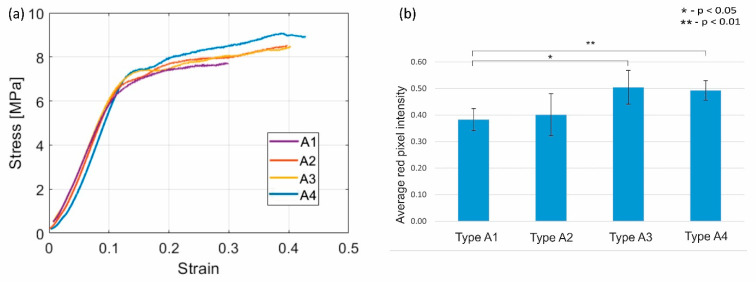
(**a**) Stress–strain curve of ABS scaffolds treated with different combinations of osteogenic agents. (**b**) Microscopy results for the corresponding Alizarin red staining imaging. The mean values of average red pixel intensities (n = 10) are represented in blue bars, and error bars represent standard deviation.

In the in vitro study of the ABS scaffolds, we find that the data collected on the mechanical properties follows normal distribution after undergoing the Shapiro–Wilk test. A one-way ANOVA test showed no significant difference in maximum compressive strength ($\sigma_{M}$), stiffness, and elasticity modulus when the scaffold groups with polylysine pre-coating were compared to the original scaffold (Types A1, A2, and A3). The Student’s *t*-test (between A1 and A4) showed us that the $\sigma_{M}$ significantly increases by 9.8% (*p* = 0.009) when CRFP is used to induce osteogenesis ($\sigma_{A4}$ = 7.71 MPa) along with G6P and AA, when compared to non-treated scaffolds ($\sigma_{A1}$ = 7.02 MPa) (Figure 5a). There was also no significant difference in the stiffness and elasticity modulus of the A1 and A4 scaffolds. Additionally, we were able to visualize and validate the calcium deposits on the surface of the implant using Alizarin red staining. We found that there is a significant increase in the calcium deposits distributed on the surface of the cylindrical implant in the case where CRFP was used with and without polylysine pre-coat (*p* = 0.012 and *p* = 0.004, respectively), (illustrated in Figure 5b).

**Figure 6:**
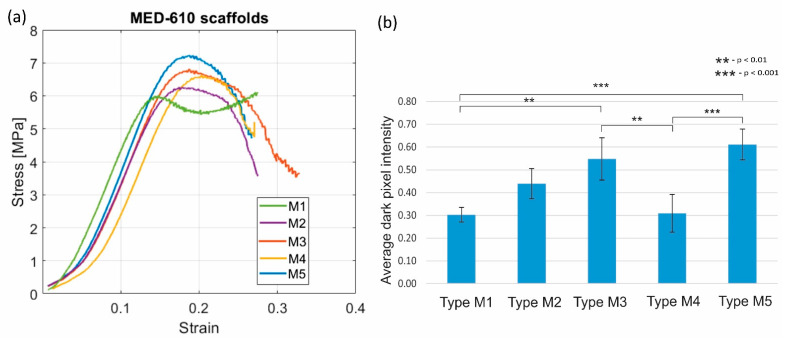
(**a**) Stress–strain curve of MED-610 scaffolds treated with different combinations of osteogenic agents. (**b**) Microscopy results for the corresponding von Kossa staining imaging. The mean values of average dark pixel intensities (n = 10) are represented in blue bars, and error bars represent standard deviation.

**Figure 7:**
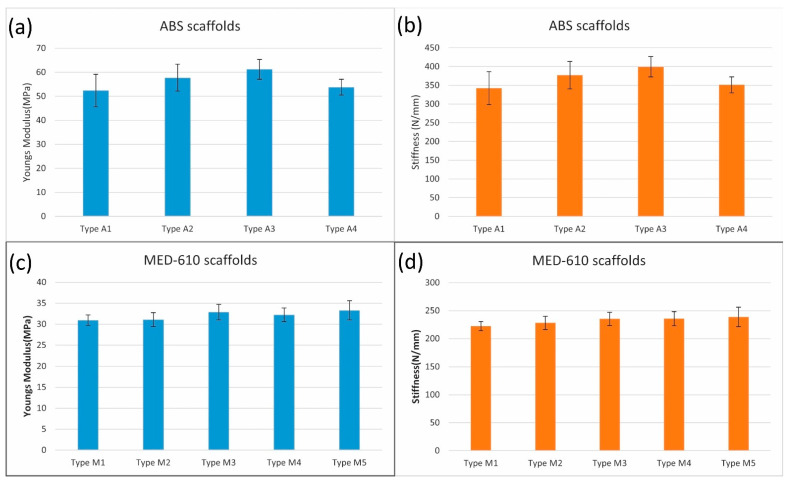
(**a**,**b**) Plots of Young’s modulus (blue) and stiffness (orange) for ABS scaffolds (n = 10) under different combinations of development from Table 1; (**c**,**d**) plots of Young’s modulus (blue) and stiffness (orange) for MED610 scaffolds (n = 10) under different combinations of development as explained in Table 2. Error bars represent the standard deviation.

In the case of MED 610 scaffolds, we found that the data collected on the compression testing follows normal distribution from the Shapiro–Wilk test. The first one-way ANOVA study showed significant differences (*p* = 0.0015) in the $\sigma_{M}$ for the three types of scaffolds (M1, M2, and M3). In the pairwise comparison by the Student’s *t*-test, we found that the strength increased significantly (*p* = 0.002) by 11.9% for scaffolds treated with CRFP ($\sigma_{M3}$ = 6.85 MPa) and coated with polylysine when compared to the plain scaffold ($\sigma_{M1}$ = 6.07 MPa). There was no significant improvement in the compressive strength when CRFP was not used. The second ANOVA study also showed a significant difference in $\sigma_{M}$ for M1, M4 and M5 scaffolds. In the pairwise comparison, we found that there was a significant 19.6% increase (*p* < 0.001) in scaffolds with CRFP treatment ($\sigma_{M5}$ = 7.26 MPa) when compared to plain scaffolds ($\sigma_{M1}$). There was no significant improvement in the compressive strength when CRFP was not used (*p* = 0.05). There was no significant increase in the stiffness and elasticity modulus even though increasing trends were observed. This is illustrated in Figure 6a. Post-compression von Kossa staining on the cylindrical scaffolds to detect calcium salts showed a significant increase in bone matrix deposition when CRFP is used with and without polylysine, (*p* = 0.0011 and *p* < 0.001, respectively). The results of microscopy are illustrated in Figure 6b. The results of stiffness and elasticity modulus for both materials are plotted in Figure 7.

**Figure 8:**
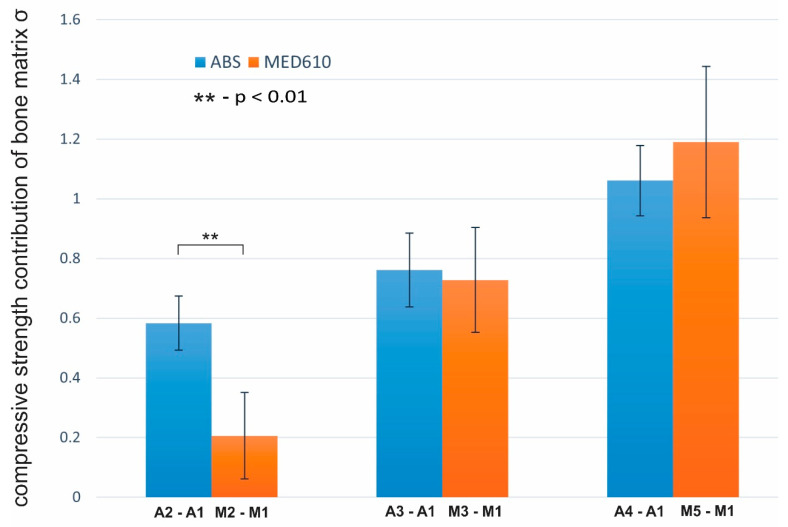
Plot of comparison of strength contributed by the bone matrix deposition on ABS material (blue) and MED610 material (orange) (n = 10). The standard deviation is represented using error bars.

On comparing the performance of ABS and MED 610 materials, we find that ABS scaffolds pre-coated with polylisine and not treated with CRFP show a significantly (*p* = 0.0085) higher contribution of strength from the bone matrix deposition ($\sigma_{A2-A1}$ = 0.58 MPa) in comparison to MED 610 scaffolds ($\sigma_{M2-M1}$ = 0.21 MPa). There is no significant change in the strength contributed by the bone matrix when ABS and MED610 were compared for the scaffolds treated with CRFP in both cases of polylysine pre-coat and no pre-coat. The results are illustrated in Figure 8.

## 4. Discussion

In this study, we worked on developing a synthetic BGS which is designed based on the cortical structure and internal trabecular structure of the bone. We used two non-biodegradable materials, vis. ABS and MED610 to fabricate 3D cylindrical implants to study their biocompatibility and osteoconductivity. These materials are fabricated with high resolution 3D printing processes to design complex structures to match the structure and mechanical properties of the bone surrounding the implantation site [42,47]. This reduces stress shielding in the implantation site [59]. Previous studies suggest that highly porous structures (>90% porosity) with a large surface area are conductive to cell proliferation on the scaffold surface [60]. However, higher porosity leads to reduction of mechanical strength of the scaffold and reduces the accuracy of consistent structural fabrication. Gregor et al. showed that similar results for cell proliferation can be obtained with BGS of lower porosities (<50% porous) [41]. Our 3D-printed scaffolds have 50% porosity to account for higher structural integrity. Moreover, the non-biodegradability of these materials helps in resolving the problem of reduced mechanical strength experienced over time by other synthetic (PLA/PCL) and ceramic materials such as tri-calcium phosphate (TCP) due to resorption [22,36].

In our in vitro studies, we established that MED610 material is osteoconductive and promotes mineralization through the evaluation of scanning electron microscopy imaging and von Kossa staining imaging studies. Other groups have shown that ABS material is biocompatible and allows for bone growth on the material surface [43]. Osteoconductivity of BGS is essential to facilitate complete integration with the surrounding bone. Moreover, these synthetic BGS do not cause immunological reactions as compared to natural BGS which are derived from other hosts [34,61]. Using MED610 will be essential in our future studies owing to its high-resolution (<16 μm) fabrication process which grants flexibility in designing more complex trabecular features, mimicking shapes of the healthy bone [62]. The compression tests show that our 3D cylindrical scaffolds developed using ABS and MED610 materials exhibit similar trends of anisotropy as that of a femur. We found that the scaffolds show the highest strength along the longitudinal axis of the cylinder (Z-axis) which mimics the longitudinal axis of the bone; whereas, the strength in the sagittal and frontal axes (X and Y directions) were considerably lower, as shown in Figure 4a,b. This behavior is similar to that of anatomical stresses of a femur bone [56,57].

$$\sigma_{Longitudinal\;axis}>\;\sigma_{Sagittal\;axis}\geq \sigma_{Frontal\;axis}$$

In our initial studies on cylindrical ABS scaffolds, we observed bone growth, osteogenesis, and osteointegration by evaluating the effect of the increase in the bone matrix on the mechanical strength of the implants. In some cases, we coated the scaffolds with polylysine before cell seeding, as polylysine is known to promote cell adherence to plastic surfaces [49]. In some cases, we added CRFP along with the osteogenic reagents as it is known to contribute to bone formation [42,44]. The analysis showed that the CRFP-treated scaffold with no pre-coating (Type A4) showed significant compressive strength increases (*p* < 0.05). We attribute this increase in strength to the formation of bone matrix deposition (shown using Alizarin red staining). The increases in the strength of other combinations of ABS scaffolds (Type A2 and Type A3) was not found to be significant.

In the studies with MED 610, we observed that the scaffolds showed a significant increase in compressive strength compared to the (Type M1) dry scaffolds (9.6% for Type M3 and 19.8% for Type M5) when they were treated with CRFP along with the osteogenic reagents. This shows that the treatment with CRFP itself plays a significant role in generating the bone matrix and improving the mineralization and biomechanical properties of the scaffolds. The stiffness and elasticity modulus for both ABS and MED610 scaffolds revealed that there was no significant increase in these properties in all of the scaffold combinations. This shows that the scaffolds treated with CRFP, which are not pre-coated, give quantitively better results.

When we compared the performance of ABS with MED610, we observed that in the case where the scaffold was pre-coated with polylysine, contribution of strength from the bone matrix decreased. However, we see that in the case where CRFP was used along with the osteoinduction reagents (G6P and AA) in both pre-coated and non-coated scaffolds, there was no significant difference between the performances of the two materials; This shows us that the bone matrix deposition on both ABS and MED610 scaffolds is nearly similar when CRFP is used during osteoinduction.

One of the limitations in the design of our scaffolds is that the cylindrical scaffold was designed by mimicking the cross-section of a femur and a cylindrical design pattern was selected for ease in our in vitro studies. Eventually, when biomimetic scaffolds are to be used for in vivo studies to replace bone stock, the scaffolds will be designed using the shape and contour of the bone surface. In this study, the pillars in the scaffolds were designed to be as thin as possible to increase the porosity of the scaffold. This pattern was selected to have a better field of view in the imaging studies and also to observe how far cells can penetrate within the grooves of the scaffolds. In the future, the thickness of the pillars can be changed to adjust for the axial stiffness of the scaffold so that it uniformly distributes stress when integrated with the healthy bone.

## 5. Conclusions

We successfully demonstrated that artificial 3D-printed scaffolds developed by seeding cells perform better biomechanically and produce more bone matrix when CRFP is used in combination with osteogenic reagents. We also found that scaffolds without pre-coating perform better in comparison to the scaffolds coated with polylysine before cell seeding. These results are essential for developing scaffolds for our future in vivo animal studies.

## Figures and Tables

**Table 1:** Different combinations of osteogenic reagents used on ABS cylindrical scaffolds for bone matrix deposition.

Type	Coating	Osteogenic Reagent
A1	Only scaffold	No reagent
A2	Polylysine coating	G6P + AA
A3	Polylysine coating	G6P + AA + CRFP
A4	No coating	G6P + AA + CRFP

**Table 2:** Different combinations of osteogenic reagents used on MED610 cylindrical scaffolds.

Type	Coating	Osteogenic Reagent
M1	Only scaffold	No reagent
M2	Polylysine coating	G6P + AA
M3	Polylysine coating	G6P + AA + CRFP
M4	No coating	G6P + AA
M5	No coating	G6P + AA + CRFP

## References

[1] de Grado G.F., Keller L., Idoux-Gillet Y., Wagner Q., Musset A.-M., Benkirane-Jessel N., Bornert F., Offner D. (2018). Bone substitutes: A review of their characteristics, clinical use, and perspectives for large bone defects management. J. Tissue Eng..

[2] Sohn H.S., Oh J.K. (2019). Review of bone graft and bone substitutes with an emphasis on fracture surgeries. Biomater. Res..

[3] Fillingham Y., Jacobs J. (2016). Bone grafts and their substitutes. Bone Jt. J..

[4] Allison D.C., Lindberg A.W., Samimi B., Mirzayan R., Menendez L.R. (2011). A Comparison of Mineral Bone Graft Substitutes for Bone Defects. Oncol. Hematol. Rev..

[5] Gazdag A.R., Lane J.M., Glaser D., Forster R.A. (1995). Alternatives to autogenous bone graft: Efficacy and indications. JAAOS-J. Am. Acad. Orthop. Surg..

[6] De Long W.G., Einhorn T.A., Koval K., McKee M., Smith W., Sanders R., Watson T. (2007). Bone grafts and bone graft substitutes in orthopaedic trauma surgery. A critical analysis. J. Bone Jt. Surg. Am. Vol..

[7] Finkemeier C.G. (2002). Bone-grafting and bone-graft substitutes. J. Bone Jt. Surg. Am. Vol..

[8] Tomford W.W. (1995). Transmission of disease through transplantation of musculoskeletal allografts. JBJS.

[9] Boyce T., Edwards J., Scarborough N. (1999). Allograft bone: The influence of processing on safety and performance. Orthop. Clin..

[10] Buck B.E., Malinin T.I., Brown M.D. (1989). Bone transplantation and human immunodeficiency virus. An estimate of risk of acquired immunodeficiency syndrome (AIDS). Clin. Orthop. Relat. Res..

[11] Mroz T.E., Joyce M.J., Steinmetz M.P., Lieberman I.H., Wang J.C. (2008). Musculoskeletal allograft risks and recalls in the United States. J. Am. Acad. Orthop. Surg..

[12] Khan S.N., Cammisa F.P., Sandhu H.S., Diwan A.D., Girardi F.P., Lane J.M. (2005). The biology of bone grafting. J. Am. Acad. Orthop. Surg..

[13] Ullmark G., Obrant K.J. (2002). Histology of impacted bone-graft incorporation. J. Arthroplast..

[14] Saikia K., Bhattacharya T., Bhuyan S., Talukdar D., Saikia S., Jitesh P. (2008). Calcium phosphate ceramics as bone graft substitutes in filling bone tumor defects. Indian J. Orthop..

[15] Gruskin E., Doll B.A., Futrell F.W., Schmitz J.P., Hollinger J.O. (2012). Demineralized bone matrix in bone repair: History and use. Adv. Drug Deliv. Rev..

[16] Wang J.C., Alanay A., Mark D., Kanim L.E., Campbell P.A., Dawson E.G., Lieberman J.R. (2007). A comparison of commercially available demineralized bone matrix for spinal fusion. Eur. Spine J..

[17] Chung H.-J., Hur J.-W., Ryu K.-S., Kim J.-S., Seong J.-H. (2016). Surgical outcomes of anterior cervical fusion using deminaralized bone matrix as stand-alone graft material: Single arm, pilot study. Korean J. Spine.

[18] Campana V., Milano G., Pagano E., Barba M., Cicione C., Salonna G., Lattanzi W., Logroscino G. (2014). Bone substitutes in orthopaedic surgery: From basic science to clinical practice. J. Mater. Sci. Mater. Med..

[19] Russell N., Walsh W.R., Lovric V., Kim P., Chen J.H., Larson M.J., Vizesi F. (2020). In-vivo Performance of Seven Commercially Available Demineralized Bone Matrix Fiber and Putty Products in a Rat Posterolateral Fusion Model. Front. Surg..

[20] Flatley T.J., Lynch K.L., Benson M. (1983). Tissue response to implants of calcium phosphate ceramic in the rabbit spine. Clin. Orthop. Relat. Res..

[21] Parikh S.N. (2002). Bone graft substitutes in modern orthopedics. Orthopedics.

[22] Hing K.A., Wilson L.F., Buckland T. (2007). Comparative performance of three ceramic bone graft substitutes. Spine J..

[23] Koshino T., Murase T., Takagi T., Saito T. (2001). New bone formation around porous hydroxyapatite wedge implanted in opening wedge high tibial osteotomy in patients with osteoarthritis. Biomaterials.

[24] Myers G.J.C., Abudu A.T., Carter S.R., Tillman R.M., Grimer R.J. (2007). Endoprosthetic replacement of the distal femur for bone tumours: Long-term results. Bone Jt. J..

[25] Shin D.S., Choong P.F., Chao E.Y., Sim F.H. (2000). Large tumor endoprostheses and extracortical bone-bridging: 28 patients followed 10–20 years. Acta Orthop. Scand..

[26] Plotz W., Rechl H., Burgkart R., Messmer C., Schelter R., Hipp E., Gradinger R. (2002). Limb salvage with tumor endoprostheses for malignant tumors of the knee. Clin. Orthop. Relat. Res..

[27] Engh C.A., Bobyn J.D., Glassman A.H. (1987). Porous-coated hip replacement. The factors governing bone ingrowth, stress shielding, and clinical results. J. Bone Jt. Surg. Br. Vol..

[28] Epari D.R., Taylor W.R., Heller M.O., Duda G.N. (2006). Mechanical conditions in the initial phase of bone healing. Clin. Biomech..

[29] Bose S., Vahabzadeh S., Bandyopadhyay A. (2013). Bone tissue engineering using 3D printing. Mater. Today.

[30] Wieding J., Fritsche A., Heinl P., Korner C., Cornelsen M., Seitz H., Mittelmeier W., Bader R. (2013). Biomechanical behavior of bone scaffolds made of additive manufactured tricalciumphosphate and titanium alloy under different loading conditions. J. Appl. Biomater. Funct. Mater..

[31] Helguero C.G., Amaya J.L., Ramirez E.A., Komatsu D.E., Kao I., Pentyala S. (2019). A manufacturing approach to functional biomimetic three-dimensional-printed bone implants. Proc. Inst. Mech. Eng. Part L J. Mater..

[32] Wong K.C. (2016). 3D-printed patient-specific applications in orthopedics. Orthop. Res. Rev..

[33] Jariwala S.H., Lewis G.S., Bushman Z.J., Adair J.H., Donahue H.J. (2015). 3D Printing of Personalized Artificial Bone Scaffolds. 3D Print. Addit. Manuf..

[34] Konopnicki S., Sharaf B., Resnick C., Patenaude A., Pogal-Sussman T., Hwang K.G., Abukawa H., Troulis M.J. (2015). Tissue-engineered bone with 3-dimensionally printed beta-tricalcium phosphate and polycaprolactone scaffolds and early implantation: An in vivo pilot study in a porcine mandible model. J. Oral Maxillofac. Surg..

[35] Yoshimoto H., Shin Y.M., Terai H., Vacanti J.P. (2003). A biodegradable nanofiber scaffold by electrospinning and its potential for bone tissue engineering. Biomaterials.

[36] Chacón J., Caminero M.A., García-Plaza E., Núnez P.J. (2017). Additive manufacturing of PLA structures using fused deposition modelling: Effect of process parameters on mechanical properties and their optimal selection. Mater. Des..

[37] Hutmacher D.W., Schantz T., Zein I., Ng K.W., Teoh S.H., Tan K.C. (2001). Mechanical properties and cell cultural response of polycaprolactone scaffolds designed and fabricated via fused deposition modeling. J. Biomed. Mater. Res..

[38] Kim J., McBride S., Tellis B., Alvarez-Urena P., Song Y.H., Dean D.D., Sylvia V.L., Elgendy H., Ong J., Hollinger J.O. (2012). Rapid-prototyped PLGA/beta-TCP/hydroxyapatite nanocomposite scaffolds in a rabbit femoral defect model. Biofabrication.

[39] Zhang B., Wang L., Song P., Pei X., Sun H., Wu L., Zhou C., Wang K., Fan Y., Zhang X. (2021). 3D printed bone tissue regenerative PLA/HA scaffolds with comprehensive performance optimizations. Mater. Des..

[40] Gunatillake P.A., Adhikari R. (2003). Biodegradable synthetic polymers for tissue engineering. Eur. Cell Mater..

[41] Gregor A., Filova E., Novak M., Kronek J., Chlup H., Buzgo M., Blahnova V., Lukasova V., Bartos M., Necas A. (2017). Designing of PLA scaffolds for bone tissue replacement fabricated by ordinary commercial 3D printer. J. Biol. Eng..

[42] Helguero C.G., Mustahsan V.M., Parmar S., Pentyala S., Pfail J.L., Kao I., Komatsu D.E., Pentyala S. (2017). Biomechanical properties of 3D-printed bone scaffolds are improved by treatment with CRFP. J. Orthop. Surg. Res..

[43] Rosenzweig D.H., Carelli E., Steffen T., Jarzem P., Haglund L. (2015). 3D-Printed ABS and PLA Scaffolds for Cartilage and Nucleus Pulposus Tissue Regeneration. Int. J. Mol. Sci..

[44] Komatsu D.E., Hadjiargyrou M., Udin S.M., Trasolini N.A., Pentyala S. (2015). Identification and Characterization of a Synthetic Osteogenic Peptide. Calcif. Tissue Int..

[45] Orcel P., Tajima H., Murayama Y., Fujita T., Krane S.M., Ogata E., Goldring S.R., Nishimoto I. (2000). Multiple domains interacting with Gs in the porcine calcitonin receptor. Mol. Endocrinol..

[46] Stratasys Ltd. Biocompatible Clear MED610 Material Data Sheet. https://www.stratasys.com/materials/search/biocompatible.

[47] Gulcan O., Gunaydin K., Tamer A. (2021). The State of the Art of Material Jetting-A Critical Review. Polymers.

[48] Udroiu R., Braga I.C. Polyjet technology applications for rapid tooling. Proceedings of the MATEC Web of Conferences.

[49] Wang T., Yang X., Qi X., Jiang C. (2015). Osteoinduction and proliferation of bone-marrow stromal cells in three-dimensional poly (epsilon-caprolactone)/ hydroxyapatite/collagen scaffolds. J. Transl. Med..

[50] Gunn N.M., Bachman M., Li G.P., Nelson E.L. (2010). Fabrication and biological evaluation of uniform extracellular matrix coatings on discontinuous photolithography generated micropallet arrays. J. Biomed. Mater. Res. A.

[51] Wang D., Christensen K., Chawla K., Xiao G., Krebsbach P.H., Franceschi R.T. (1999). Isolation and characterization of MC3T3-E1 preosteoblast subclones with distinct in vitro and in vivo differentiation/mineralization potential. J. Bone Miner. Res. Off. J. Am. Soc. Bone Miner. Res..

[52] Tenenbaum H.C., Torontali M., Sukhu B. (1992). Effects of bisphosphonates and inorganic pyrophosphate on osteogenesis in vitro. Bone.

[53] Franceschi R.T., Iyer B.S., Cui Y. (1994). Effects of ascorbic acid on collagen matrix formation and osteoblast differentiation in murine MC3T3-E1 cells. J. Bone Miner. Res. Off. J. Am. Soc. Bone Miner. Res..

[54] USP United States Pharmacopeia and National Formulary. https://www.usp.org/reference-standards.

[55] ISO 604: 2002 (2002). Plastics-Determination of Compressive Properties.

[56] Zhao S., Arnold M., Ma S., Abel R.L., Cobb J.P., Hansen U., Boughton O. (2018). Standardizing compression testing for measuring the stiffness of human bone. Bone Jt. Res..

[57] Novitskaya E., Chen P.Y., Lee S., Castro-Cesena A., Hirata G., Lubarda V.A., McKittrick J. (2011). Anisotropy in the compressive mechanical properties of bovine cortical bone and the mineral and protein constituents. Acta Biomater..

[58] Hasegawa T., Miwa M., Sakai Y., Niikura T., Lee S., Oe K., Iwakura T., Kurosaka M., Komori T. (2010). Efficient cell-seeding into scaffolds improves bone formation. J. Dent. Res..

[59] Mi Z.R., Shuib S., Hassan A., Shorki A., Ibrahim M. (2007). Problem of stress shielding and improvement to the hip Implat designs: A review. J. Med. Sci..

[60] Polo-Corrales L., Latorre-Esteves M., Ramirez-Vick J.E. (2014). Scaffold design for bone regeneration. J. Nanosci. Nanotechnol..

[61] DeLustro F., Dasch J., Keefe J., Ellingsworth L. (1990). Immune responses to allogeneic and xenogeneic implants of collagen and collagen derivatives. Clin. Orthop. Relat. Res..

[62] Chen Y.W., Fang H.Y., Shie M.Y., Shen Y.F. (2019). The mussel-inspired assisted apatite mineralized on PolyJet material for artificial bone scaffold. Int. J. Bioprint..

